# Effects of Thermal Aging and Subsequent Autoclave Treatment on the Phytochemical Fingerprint, Antioxidant Capacity, and In Vitro Carbohydrate-Hydrolyzing Enzyme Inhibitory Activity of Lychee Pulp Extracts

**DOI:** 10.3390/plants15152290

**Published:** 2026-07-26

**Authors:** Churdsak Jaikang, Supakit Chaipoot, Hataichanok Chuljerm, Kanokwan Kulprachakarn, Anupon Iadnut, Giatgong Konguthaithip, Narongsuk Munkong, Surasawadee Somnuk, Kongsak Boonyapranai, Sakaewan Ounjaijean, Wason Parklak

**Affiliations:** 1 Department of Forensic Medicine, Faculty of Medicine, Chiang Mai University, Chiang Mai 50200, Thailand; churdsak.j@cmu.ac.th (C.J.); kongkiat.shang@gmail.com (G.K.); 2 Metabolomic Research Group for Forensic Medicine and Toxicology, Department of Forensic Medicine, Faculty of Medicine, Chiang Mai University, Chiang Mai 50200, Thailand; 3 Multidisciplinary Research Institute, Chiang Mai University, Chiang Mai 50200, Thailand; supakit.ch@cmu.ac.th; 4 School of Health Sciences Research, Research Institute for Health Sciences, Chiang Mai University, Chiang Mai 50200, Thailand; hataichanok.ch@cmu.ac.th (H.C.); kanokwan.kul@cmu.ac.th (K.K.); kongsak.b@cmu.ac.th (K.B.); sakaewan.o@cmu.ac.th (S.O.); 5 Faculty of Medicine, Nation University, Chiang Mai 50210, Thailand; anupon_iad@nation.ac.th; 6 Department of Pathology, School of Medicine, University of Phayao, Phayao 56000, Thailand; narongsuk.mu@up.ac.th; 7 Department of Sports Science, Faculty of Sports and Health Science, Kasetsart University, Kamphaeng Saen Campus, Nakhon Pathom 73140, Thailand; surasawadee.so@ku.th

**Keywords:** lychee, thermal aging, autoclave treatment, ^1^H NMR, phytochemical fingerprint, antioxidant activity, α-amylase inhibition, α-glucosidase inhibition, cytotoxicity

## Abstract

Background: This study evaluated the effects of aging and subsequent autoclave treatment on the proton nuclear magnetic resonance (^1^H NMR) spectral-feature profile and biological activities of lychee pulp extracts. Methods: Dried lychee (DL), aged lychee (AL), and autoclave-treated aged lychee (ATAL) extracts were compared using ^1^H NMR spectroscopy, total phenolic content (TPC), total flavonoid content (TFC), antioxidant assays, carbohydrate-hydrolyzing enzyme inhibitory assays, and endothelial cell viability. Results: Sixteen phytochemical spectral features were putatively annotated, and their relative signal intensities differed among the extracts (*p* < 0.001). Relative to DL, ATAL exhibited higher signal intensities for most features, including taxifolin (8.32-fold) and procyanidin B2 (7.44-fold), whereas epicatechin was particularly prominent in AL (12.31-fold). TPC increased from 0.53 ± 0.07 mg gallic acid equivalents (GAE)/g extract in DL to 8.39 ± 0.19 and 9.07 ± 0.10 mg GAE/g extract in AL and ATAL, respectively. AL and ATAL also showed higher TFC and antioxidant activities than DL. The α-amylase IC_50_ values were 247.00 ± 45.00, 129.17 ± 8.56, and 126.27 ± 3.90 mg/mL for DL, AL, and ATAL, respectively, while the corresponding α-glucosidase IC_50_ values were 187.93 ± 8.20, 66.71 ± 6.84, and 50.30 ± 2.72 mg/mL. No significant reduction in endothelial cell viability was detected after multiplicity adjustment. Exploratory principal component analysis (PCA) explained 99.8% of the total variance and revealed distinct processing-associated spectral-feature profiles. Conclusions: Aging followed by autoclave treatment enhanced the phytochemical profile, antioxidant activity, and in vitro carbohydrate-hydrolyzing enzyme inhibition of lychee pulp extracts.

## 1. Introduction

Lychee (*Litchi chinensis* Sonn.) is an economically important tropical fruit rich in sugars, organic acids, vitamin C, and phenolic compounds [1]. The Hong Huay cultivar is widely cultivated in northern Thailand and has been used for fresh consumption and processed products [2]. Lychee-derived phenolics, including gallic acid, catechin, epicatechin, procyanidins, quercetin, and rutin, have been associated with antioxidant, anti-inflammatory, and antidiabetic activities [1,2,3,4]. These compounds may contribute to metabolic health by reducing oxidative stress and modulating enzymes involved in carbohydrate digestion [5,6].

Postprandial hyperglycemia is mainly driven by the rapid hydrolysis of dietary starch into absorbable glucose. Pancreatic α-amylase hydrolyzes starch into smaller oligosaccharides, while intestinal α-glucosidase further converts these products into glucose before absorption [7]. Therefore, inhibition of these enzymes can delay carbohydrate digestion, slow glucose release, and attenuate postprandial blood glucose elevation [7]. Phenolic compounds may inhibit α-amylase and α-glucosidase through hydrogen bonding, hydrophobic interactions, and binding to catalytic or allosteric sites of the enzymes, leading to reduced enzyme–substrate interaction [8]. In addition, antioxidant phenolics may indirectly support glucose homeostasis by reducing oxidative stress, which is closely linked to insulin resistance and β-cell dysfunction [5,8].

Recent advances in food processing have focused not only on extending shelf life but also on preserving or enhancing the recovery of bioactive constituents from plant materials. Emerging approaches, including high-pressure processing, pulsed electric fields, ultrasound, microwave-assisted processing, and controlled fermentation, can improve phytochemical extractability by modifying plant tissues and facilitating mass transfer [9,10,11,12,13,14]. Nevertheless, the effectiveness of these technologies depends on the food matrix, processing conditions, and chemical stability of the target compounds [13,15]. Conventional thermal processes also remain relevant because they are widely available and comparatively straightforward to implement. Although heating may degrade thermolabile constituents [16], controlled thermal processing can also release matrix-associated phenolics, transform existing compounds, and generate Maillard reaction products with antioxidant properties [14]. Recent literature discusses applications of emerging nonthermal processing technologies and combinations of pulsed electric fields and ultrasound for improving phytochemical recovery [17].

Among thermal processing approaches, controlled aging can modify phenolic extractability and promote Maillard-related changes in plant materials. Our previous study on dried Hong Huay lychee pulp demonstrated that aging at 60–70 °C increased total phenolic content, total flavonoid content, and antioxidant activity. Based on these findings, aging at 70 °C for 20 days was selected for the present study [2]. The observed changes may be associated with the release of matrix-associated phenolics, improved phenolic extractability, and the formation of Maillard reaction products with antioxidant properties. Nevertheless, excessive heating may degrade thermolabile phenolics, sugars, and amino acids, emphasizing the importance of carefully controlled processing conditions [16].

Autoclave treatment is a moist-heat process in which food or plant materials are exposed to saturated steam at elevated temperature and pressure. Such treatment can modify plant tissue structure and the extractability of matrix-associated phytochemicals. Depending on the treatment conditions and food matrix, moist heating may facilitate the release or transformation of certain phenolic constituents [18,19], whereas prolonged exposure to high temperatures may also cause oxidation, polymerization, epimerization, or degradation of thermolabile compounds [16]. Previous studies have reported changes in phenolic profiles and antioxidant properties following autoclave treatment of plant-derived materials [20]. In lychee, thermal processing at 121 °C has been shown to alter phenolic composition and antioxidant properties in both lychee pulp and lychee juice [21]. Autoclave treatment has also modified phenolic composition and antioxidant activity in other plant-derived materials, including Opuntia fruit [22] and brewers’ spent grain [23].

Nevertheless, the effects of applying autoclave treatment after prolonged thermal aging on the chemical fingerprint and in vitro biological properties of lychee pulp remain poorly understood. Previous studies have examined either thermal aging or high-temperature processing of lychee; however, the successive application of these treatments has not been investigated in Hong Huay lychee. Therefore, this study aimed to compare dried lychee (DL), aged lychee (AL), and autoclave-treated aged lychee (ATAL) in terms of their chemical fingerprints, total phenolic and flavonoid contents, antioxidant activities, and inhibitory activities against α-amylase and α-glucosidase. Chemical fingerprinting was performed using ^1^H NMR spectroscopy, while cytotoxicity toward endothelial cells was evaluated to provide preliminary in vitro safety information relevant to potential functional food applications.

## 2. Results

### 2.1. Comparative ^1^H NMR Spectral Profiling and Putative Annotation of Processed Lychee Pulp Extracts

The extraction yields were 12.29 ± 0.84%, 13.09 ± 0.53%, and 13.16 ± 0.46% for DL, AL, and ATAL, respectively. No significant difference in extraction yield was observed among the three treatments (one-way ANOVA, exact *p* = 0.250).

**Figure 1:**
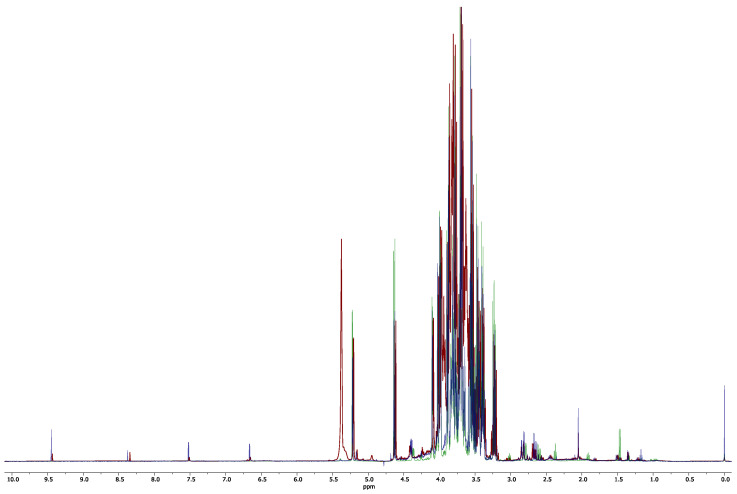
Overlay of representative ^1^H NMR spectra of dried lychee extract (DL, green), aged lychee extract (AL, red), and autoclave-treated aged lychee (ATAL, blue).

Representative overlaid ^1^H NMR spectra of the DL, AL, and ATAL extracts are presented in Figure 1. Sixteen phytochemical spectral features were putatively annotated based on their diagnostic ^1^H NMR signals and comparison with database and published spectral information (Table 1). The relative signal intensities of all 16 putatively annotated spectral features differed significantly among the DL, AL, and ATAL extracts (one-way ANOVA, all *p* < 0.001; Table 1). For each spectral feature, the mean signal intensity of the DL group was normalized to 1.00, while the AL and ATAL values were expressed as fold differences relative to DL. Exact omnibus *p*-values, Tukey-adjusted pairwise *p*-values, and simultaneous 95% confidence intervals are provided in Supplementary Table S1.

The ATAL extract exhibited the highest relative signal intensities for most of the putatively annotated features. Particularly pronounced increases relative to DL were observed for taxifolin (8.32-fold), procyanidin B2 (7.44-fold), meloside (7.10-fold), p-coumaric acid (6.46-fold), quercetin (5.61-fold), epicatechin (5.18-fold), and epigallocatechin gallate (EGCG; 4.86-fold). ATAL also showed higher signal intensities of quercetin 4′-glycoside (3.67-fold), delphinidin 3-glucoside (3.42-fold), isoquercitrin (3.34-fold), cyanidin (2.53-fold), catechin (2.51-fold), and kaempferol (2.48-fold) than DL. Tukey’s HSD analysis confirmed that these ATAL values were significantly higher than the corresponding DL values (adjusted *p* < 0.05).

The AL extract displayed a distinct phytochemical signal pattern. Epicatechin exhibited the largest relative signal intensity in AL, reaching 12.31-fold that of DL and exceeding the value observed in ATAL. AL also showed substantially higher signals for p-coumaric acid (6.34-fold), gallic acid (5.45-fold), caffeic acid (4.60-fold), and EGCG (2.39-fold) than DL. Gallic acid, caffeic acid, and epicatechin signals were significantly higher in AL than in ATAL, whereas the difference in p-coumaric acid between AL and ATAL was not significant. In contrast, AL produced lower relative signal intensities for rutin (0.46-fold), isoquercitrin (0.55-fold), quercetin 4′-glycoside (0.73-fold), cyanidin (0.51-fold), and delphinidin 3-glucoside (0.31-fold) than DL. No significant differences between DL and AL were observed for catechin, meloside, quercetin, procyanidin B2, or taxifolin.

### 2.2. Total Phenolic and Flavonoid Contents and Antioxidant Activities of Lychee Pulp Extracts

The total phenolic content (TPC), total flavonoid content (TFC), and antioxidant activities of the lychee pulp extracts are presented in Table 2. Significant overall differences among the extracts were observed for TPC, TFC, DPPH radical scavenging activity, ABTS radical cation scavenging activity, and FRAP (all *p* < 0.001). Exact omnibus *p*-values and Tukey-adjusted pairwise *p*-values, together with simultaneous 95% confidence intervals (95% CIs) for the pairwise mean differences, are provided in Supplementary Table S2.

DL showed the lowest TPC, at 0.53 ± 0.07 mg GAE/g extract (95% CI: 0.37–0.69), followed by AL at 8.39 ± 0.19 mg GAE/g extract (95% CI: 7.92–8.86) and ATAL at 9.07 ± 0.10 mg GAE/g extract (95% CI: 8.84–9.31). Significant differences were observed among all three extracts. TFC values were 0.20 ± 0.03, 2.89 ± 0.07, and 3.22 ± 0.05 mg QE/g extract for DL, AL, and ATAL, respectively, with significant differences among the three extracts.

DPPH radical scavenging activity was significantly lower in DL than in AL and ATAL, while no significant difference was observed between AL and ATAL. ABTS radical cation scavenging activity differed significantly among all three extracts, with the highest value observed in ATAL, followed by AL and DL. FRAP was significantly lower in DL than in AL and ATAL, whereas no significant difference was observed between AL and ATAL.

### 2.3. Carbohydrate-Hydrolyzing Enzyme Inhibitory Activities of Lychee Pulp Extracts

**Figure 2:**
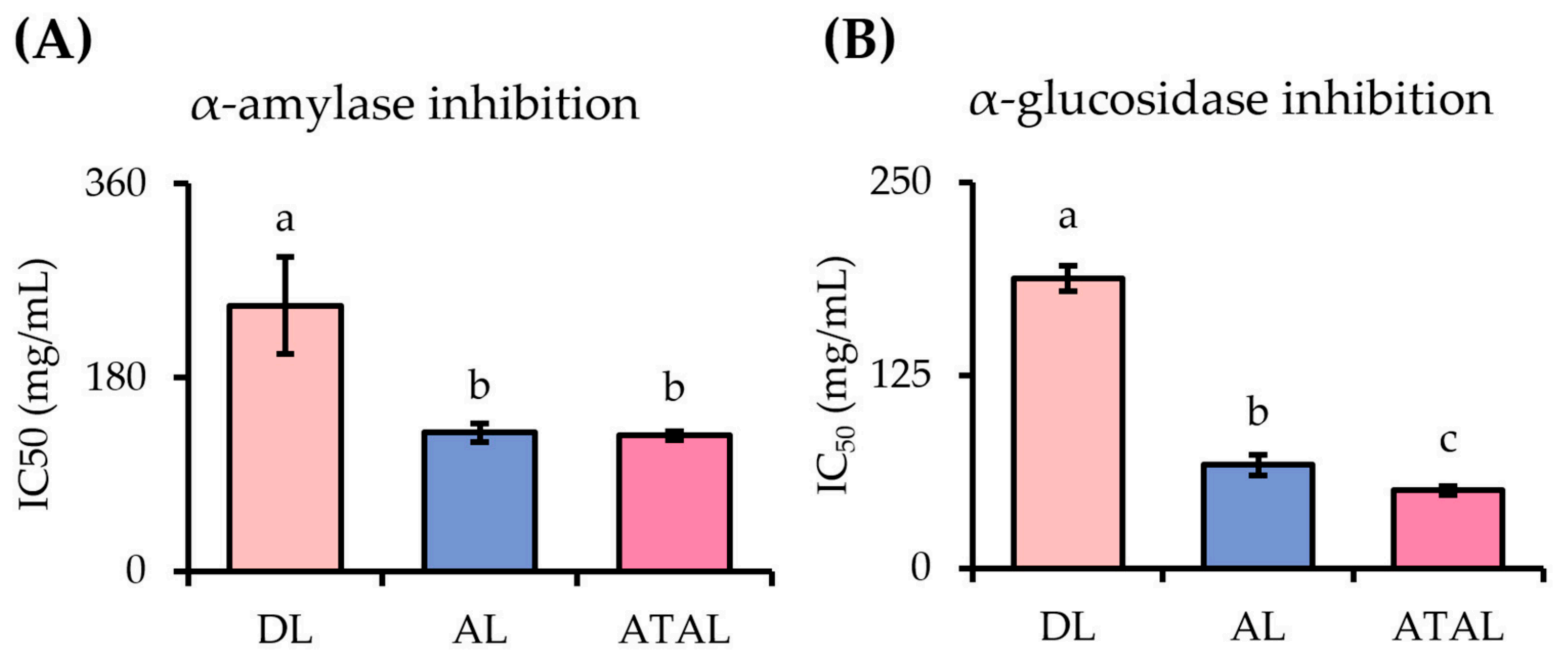
In vitro inhibitory activities of lychee pulp extracts against (**A**) α-amylase and (**B**) α-glucosidase, expressed as half-maximal inhibitory concentration (IC_50_) values. Bars represent the mean ± SD of three independent extraction batches (*n* = 3). Different lowercase letters indicate significant differences among extracts within the same enzyme assay according to one-way ANOVA followed by Tukey’s HSD test (adjusted *p* < 0.05). DL, dried lychee extract; AL, aged lychee extract; ATAL, autoclave-treated aged lychee extract.

The inhibitory activities of the lychee pulp extracts against α-amylase and α-glucosidase, expressed as IC_50_ values, are presented in Figure 2. Significant overall differences among the extracts were observed for both enzyme assays. Exact omnibus *p*-values, Tukey-adjusted pairwise *p*-values, and simultaneous 95% confidence intervals (95% CIs) for the pairwise mean differences are provided in Supplementary Table S2.

For α-amylase inhibition, DL exhibited a significantly higher IC_50_ value (247.00 ± 45.00 mg/mL) than AL (129.17 ± 8.56 mg/mL) and ATAL (126.27 ± 3.90 mg/mL). No significant difference was observed between AL and ATAL. For α-glucosidase inhibition, the IC_50_ values were 187.93 ± 8.20, 66.71 ± 6.84, and 50.30 ± 2.72 mg/mL for DL, AL, and ATAL, respectively. All three extracts differed significantly from one another, with ATAL exhibiting the lowest IC_50_ value.

### 2.4. Effects of Lychee Pulp Extracts on Endothelial Cell Viability

**Figure 3:**
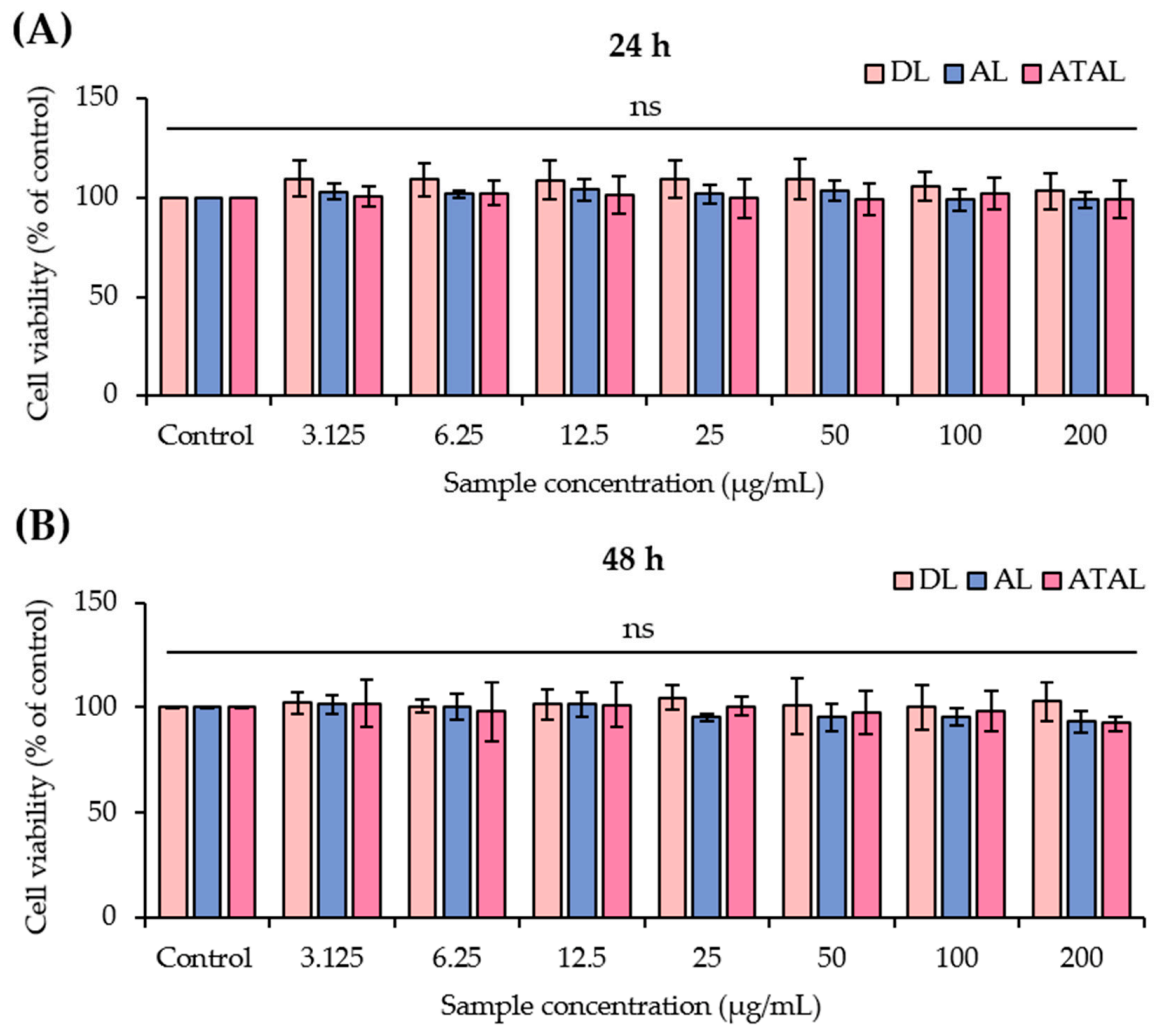
Effects of lychee pulp extracts on EA.hy926 endothelial cell viability after exposure for (**A**) 24 h and (**B**) 48 h. Values represent the mean ± SD of three independent experiments. Four technical wells were averaged within each independent experiment. Comparisons with the normalized control value of 100% were performed using two-sided one-sample *t*-tests with Holm adjustment for multiple comparisons. ns, not significant (Holm-adjusted *p* > 0.05).

After 24 and 48 h of treatment, none of the DL, AL, or ATAL extracts at concentrations ranging from 3.125 to 200 µg/mL produced a statistically significant change in EA.hy926 cell viability compared with the corresponding normalized control after adjustment for multiple comparisons (all Holm-adjusted *p* > 0.05; Figure 3). Cell viability remained above approximately 92% across the tested conditions. Exact *p*-values and 95% confidence intervals are provided in Supplementary Table S3.

### 2.5. Exploratory Multivariate Analysis of ^1^H NMR Spectral-Feature Profiles

**Figure 4:**
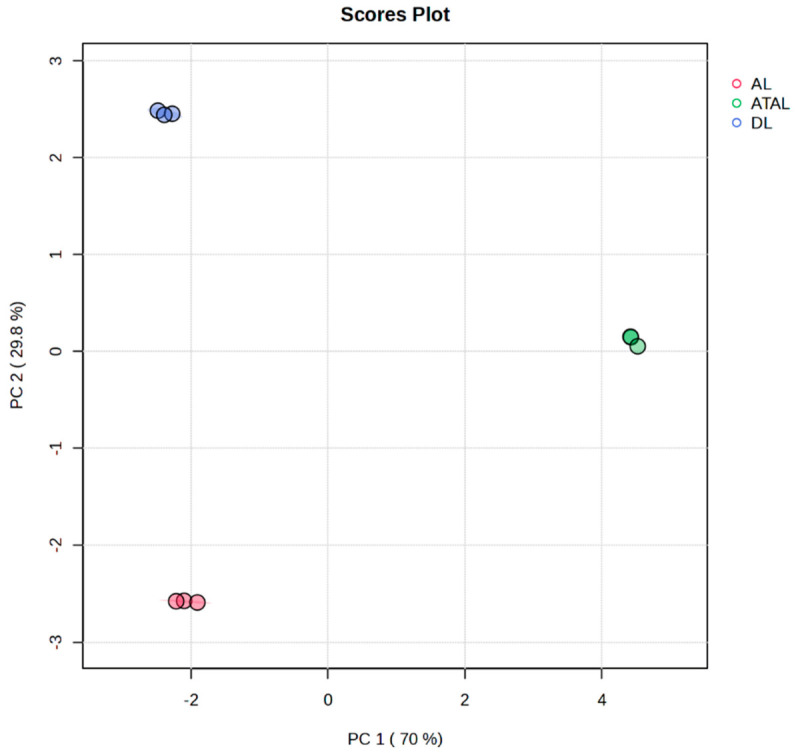
Exploratory principal component analysis of the relative ^1^H NMR spectral-feature profiles of lychee pulp extracts. The PCA score plot was generated from the relative signal intensities of 16 putatively annotated ^1^H NMR spectral features. Data were log_2_-transformed, mean-centered, and autoscaled to unit variance before analysis. PC1 and PC2 explained 70.0% and 29.8% of the total variance, respectively. Each point represents one independent replicate (*n* = 3 per processing condition). DL, dried lychee extract; AL, aged lychee extract; ATAL, autoclave-treated aged lychee extract.

Exploratory principal component analysis was performed using the log_2_-transformed and autoscaled relative signal intensities of 16 putatively annotated ^1^H NMR spectral features. The first two principal components explained 99.8% of the total variance, with PC1 and PC2 accounting for 70.0% and 29.8%, respectively. The score plot revealed distinct processing-associated spectral-feature patterns among the DL, AL, and ATAL extracts (Figure 4), while the three replicates within each processing condition showed closely grouped scores. ATAL was separated from DL and AL primarily along PC1, whereas DL and AL were differentiated mainly along PC2. Consistent processing-associated patterns were also visualized in the hierarchical clustering heatmap of the 16 putatively annotated ^1^H NMR spectral features (Figure S1).

## 3. Discussion

The ^1^H NMR analysis indicated that aging and subsequent autoclave treatment modified the spectral-feature profile of lychee pulp extracts. Although DL, AL, and ATAL exhibited broadly comparable spectral patterns, differences were observed in the relative intensities of several diagnostic signals. Sixteen spectral features were putatively annotated and descriptively grouped into seven phytochemical classes, including phenolic acids, flavan-3-ols, proanthocyanidins, flavanonols, flavonols, flavonoid glycosides, and anthocyanins. These annotations are consistent with previous reports identifying polyphenols, particularly flavan-3-ol monomers and dimers, as important phytochemical constituents of lychee [2,24,25]. However, the present NMR results represent relative signal intensities rather than absolute concentrations; therefore, they should be interpreted as processing-associated spectral differences rather than quantitative measurements of individual phytochemicals.

The putatively annotated signals of gallic acid, caffeic acid, and p-coumaric acid were higher in AL and ATAL than in DL. This pattern may be associated with the effects of prolonged thermal aging on plant cell structures and phenolic conjugates [14]. Phenolic acids can occur in free forms or as bound forms associated with cell-wall components or phenolic polymers [26]. Thermal treatment may promote matrix softening, hydrolysis of conjugated forms, and increased solvent accessibility, thereby increasing the detectable signals of released phenolic constituents. Gallic acid, for example, may be released from hydrolysable tannins or galloylated phenolic compounds under hydrolytic conditions [27]. Nevertheless, because cell-wall structure and phenolic conjugates were not directly examined, these mechanisms remain plausible explanations rather than experimentally demonstrated pathways.

Different processing-associated patterns were observed among the flavan-3-ol-related signals. Epicatechin exhibited the highest relative signal intensity in AL, reaching 12.31-fold that of DL, whereas catechin and epigallocatechin gallate showed higher relative signal intensities in ATAL. Flavan-3-ols are sensitive to temperature, moisture, oxygen, and processing duration and may undergo release from the food matrix, epimerization, oxidation, polymerization, or degradation [28,29]. Heating may also promote interconversion among catechin-related and gallated structures [30]. Accordingly, the differences among the epicatechin-, catechin-, and epigallocatechin gallate-related signals may reflect the net balance among release, transformation, and degradation. The pronounced increase in the procyanidin B2-related signal in ATAL may likewise indicate improved detectability of proanthocyanidin-related constituents; however, confirmation using an orthogonal analytical technique would be required to distinguish release from thermal transformation.

The treatment applied in this study is more accurately described as autoclave treatment rather than conventional steam explosion. The aged lychee material was heated at 121 °C for 30 min under pressurized moist-heat conditions, followed by pressure release; however, reactor-specific steam-explosion parameters and decompression kinetics were not characterized. Pressurized autoclave treatment can modify plant matrices and macromolecular structures, potentially increasing solvent accessibility to intracellular and cell-wall-associated constituents. Previous studies have shown that autoclave treatment can alter fiber structure, soluble components, phenolic composition, and antioxidant properties, although the direction and magnitude of these changes depend on the plant material and processing conditions [19,20,21,22,23,31]. This may partly explain the higher relative signals of taxifolin, procyanidin B2, meloside, quercetin, quercetin 4′-glycoside, and other flavonoid-related features in ATAL. Nevertheless, heat may simultaneously promote hydrolysis, interconversion, oxidation, or degradation [16,32]; therefore, the observed signals represent the net outcome of several competing processes.

The higher cyanidin- and delphinidin 3-glucoside-related signals in ATAL should also be interpreted cautiously. Anthocyanins are generally heat-sensitive, and their stability depends on pH, glycosylation, acylation, oxygen exposure, temperature, and treatment duration [33,34]. Thus, the higher signals do not necessarily indicate preservation or increased absolute concentrations of native anthocyanins. They may instead reflect improved release from the processed matrix, formation of related transformation products, or signal overlap within the crowded aromatic and sugar regions of the one-dimensional spectra. Overall, AL was characterized by a pronounced epicatechin-related signal, whereas ATAL exhibited higher relative signals for several flavonoid-, flavonoid glycoside-, proanthocyanidin-, and anthocyanin-related features.

The NMR spectral changes were accompanied by increases in TPC, TFC, and antioxidant activities. Compared with DL, both AL and ATAL exhibited higher TPC and TFC together with greater DPPH, ABTS, and FRAP activities. In contrast, extraction yield did not differ significantly among DL, AL, and ATAL, with values of 12.29 ± 0.84%, 13.09 ± 0.53%, and 13.16 ± 0.46%, respectively (*p* = 0.250). This finding suggests that processing primarily altered the chemical and functional characteristics of the recovered extracts rather than substantially increasing total extract recovery. Nevertheless, TPC and TFC assays measure collective reagent-reactive capacity and are not specific measurements of individual compounds. Their agreement with the NMR signal patterns therefore provides complementary evidence of processing-associated changes but does not confirm the identities or absolute concentrations of individual phytochemicals.

Phenolic acids and flavonoids can contribute to antioxidant activity through electron or hydrogen-atom donation, radical stabilization, and metal chelation [35,36]. Structural characteristics—including the number and position of hydroxyl groups, conjugation, glycosylation, and polymerization—can influence these activities [37]. Recent studies similarly emphasize that plant polyphenols exhibit diverse biological and antioxidant properties, while their activities depend strongly on chemical structure, concentration, and bioavailability [38]. The higher TPC, TFC, and antioxidant activities of AL and ATAL are therefore compatible with their altered phenolic-related signal profiles. The numerically higher FRAP value in AL than in ATAL, despite their comparable TPC and TFC values, further indicates that antioxidant activity depends on qualitative composition as well as total reagent-reactive content.

AL and ATAL also exhibited lower α-amylase and α-glucosidase IC_50_ values than DL. Phenolic acids, flavan-3-ols, flavonols, and proanthocyanidins have been reported to interact with carbohydrate-hydrolyzing enzymes, potentially affecting catalytic-site accessibility and enzyme conformation [39,40]. The concurrent changes in several phenolic-related signals therefore provide a plausible chemical context for the improved inhibitory activities. However, the present study did not directly test compound–enzyme interactions, and the revised multivariate analysis did not evaluate correlations between phytochemical signals and biological activities. The observed enzyme inhibition should consequently be interpreted as a property of the whole extracts rather than as evidence of activity by specific compounds.

Importantly, the α-amylase and α-glucosidase IC_50_ values remained in the range of tens to hundreds of milligrams per milliliter. Although aging and autoclave treatment improved the activities relative to DL, the extracts were substantially less potent than acarbose and many medicinal-plant-derived inhibitors. These assays demonstrate only in vitro inhibition of carbohydrate-hydrolyzing enzymes and do not establish antidiabetic efficacy. Translation to a functional-food application would depend on achievable serving concentrations, gastrointestinal stability, bioaccessibility, interactions with the food matrix, metabolism, and tolerability. Broader evidence concerning plant-derived bioactive compounds likewise indicates that enzyme inhibition is only one potential mechanism relevant to glucose regulation and that cell-based, animal, and ultimately clinical evidence is required before antidiabetic effects can be inferred [41].

Exploratory PCA provided an overall visualization of the processing-associated ^1^H NMR spectral profiles. The analysis was restricted to the 16 putatively annotated spectral features and did not include TPC, TFC, antioxidant activities, or enzyme-inhibition data. PC1 and PC2 explained 70.0% and 29.8% of the total variance, respectively. ATAL was separated from DL and AL mainly along PC1, whereas DL and AL were differentiated along PC2. The close grouping of the three independent extraction replicates within each treatment indicated consistent spectral-feature patterns. Similar patterns were visualized in the hierarchical clustering heatmap (Figure S1).

The cell-viability assay provided preliminary information on cytocompatibility under the tested conditions. Mean viability remained above 92% across all extract concentrations and exposure times, and no condition differed significantly from the corresponding normalized control after Holm adjustment. The previously observed reduction at 200 µg/mL ATAL after 48 h was therefore not significant after correction for multiple comparisons. These findings indicate an absence of detectable cytotoxicity in EA.hy926 endothelial cells under the tested conditions; however, they do not establish general safety following oral consumption. Additional studies using intestinal and hepatic models, repeated exposure, and physiologically relevant concentrations would be needed for a broader safety assessment.

From a practical perspective, although aging and subsequent autoclave treatment introduced additional processing steps, they were associated with modest numerical increases in extraction yield, from 12.29% in DL to 13.09% in AL and 13.16% in ATAL, although these differences were not statistically significant (*p* = 0.250). These treatments also enhanced TPC, TFC, antioxidant activities, and in vitro carbohydrate-hydrolyzing enzyme inhibition, suggesting that their principal value lies in improving the functional characteristics of the extracts rather than substantially increasing material recovery. Nevertheless, scale-up would require optimization of steam and energy consumption, cooling-water operation, processing time, throughput, temperature uniformity, and product quality [42,43,44]. The industrial feasibility of the present process and its superiority over conventional heating also remain to be established.

Several analytical limitations should be considered. The 16 features were putatively annotated using one-dimensional ^1^H NMR chemical shifts, signal multiplicities, database entries, and published literature; therefore, closely related flavonoids and glycosides may remain unresolved in overlapping spectral regions. Because the acquisition method was not validated for quantitative NMR, the results are presented as relative signal intensities (fold of DL) rather than absolute concentrations. Confirmation using authentic standards, 2D NMR, LC–MS/MS, or HPLC-DAD/MS would strengthen future compound-level characterization [45].

## 4. Materials and Methods

### 4.1. Preparation of Lychee Samples and Extracts

Fresh lychee fruits (*Litchi chinensis* Sonn. var. Hong Huay) were obtained from Chiang Mai Province, Thailand, during the harvesting season (April–May 2025).

#### 4.1.1. Dried Lychee (DL)

Whole fresh lychee fruits were dried using a hot-air oven (model UNE 600, Memmert GmbH + Co. KG, Schwabach, Germany) at 60 °C until the moisture content reached approximately 16–18%, according to the method described by Chaipoot et al. [2]. The dried whole-fruit samples were designated as DL and served as the control sample.

#### 4.1.2. Aged Lychee (AL)

Aged lychee was prepared according to the moist–dry–heating system described by Chaipoot et al. [2]. Briefly, 300 g of dried whole lychee fruits (DL) were placed in desiccators maintained at 75% relative humidity using a saturated NaCl solution and incubated in an incubator (DAIHAN Scientific Co., Ltd., Wonju, Republic of Korea) at 70 °C for 20 days. The resulting samples were designated as AL.

#### 4.1.3. Autoclave-Treated Aged Lychee (ATAL)

The aged lychee material was subsequently treated in a laboratory autoclave (HICLAVE, model HVN-85, Hirayama Manufacturing Corp., Saitama, Japan) at 121 °C under saturated steam at approximately 0.10 MPa gauge pressure for 30 min. After completion of the treatment cycle, the chamber pressure was released according to the standard operating procedure of the autoclave. The treated material was allowed to cool to room temperature and was designated as autoclave-treated aged lychee (ATAL).

#### 4.1.4. Preparation of Lychee Pulp Extracts

**Figure 5:**
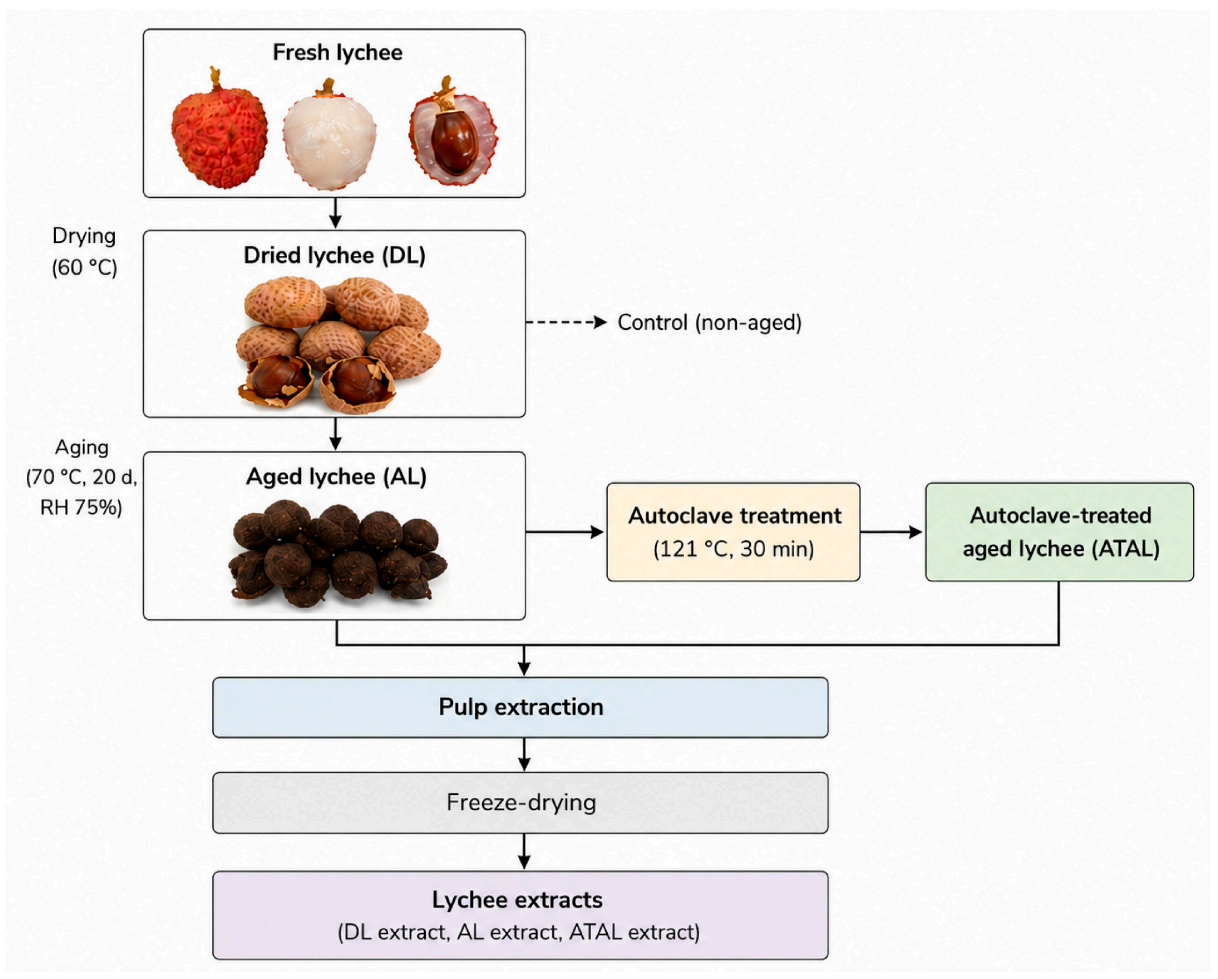
Summarizes the preparation process of DL, AL, and ATAL extracts.

For extract preparation, DL, AL, and ATAL samples were manually dehulled, and only the pulp portion was used for extraction. Lychee pulp (100 g) was mixed with distilled water at a ratio of 1:5 (*w*/*v*) and blended using a hand mixer (800 W, Philips, Bangkok, Thailand) for 2 min. The homogenate was subjected to ultrasonic extraction for 10 min at 25 °C and centrifuged at 8000 rpm for 15 min. The supernatant was collected and filtered through Whatman No. 4 filter paper (pore size 20–25 μm). The filtrate was subsequently freeze-dried (lyophilized) to obtain powdered lychee pulp extracts according to the method described by Somjai et al. [46], with slight modifications. The resulting extracts were referred to as DL extract, AL extract, and ATAL extract, respectively, and were stored in amber bottles at −20 °C until further analyses. The overall workflow for the preparation of lychee extracts is summarized in Figure 5.

For each treatment (DL, AL, and ATAL), three separate portions of the corresponding processed material were independently extracted and treated as independent extraction replicates (*n* = 3). The extraction yield of each replicate was calculated as follows: extraction yield (%) = (mass of the dried extract/mass of the starting material used for extraction) × 100. Replicate measurements or wells performed using the same extract within each assay were considered technical replicates and were averaged to obtain one value per independent extract for statistical analysis. The number of technical replicates is specified in the corresponding assay subsection.

### 4.2. ^1^H NMR Spectral Profiling

^1^H NMR spectral profiling was performed according to a previously described method [47], with modifications. Freeze-dried extracts from three independent extractions per treatment were weighed and dissolved in D_2_O containing trimethylsilylpropionic-2,2,3,3-d_4_ acid sodium salt (TSP-d_4_) as the chemical-shift reference. After vortex mixing and centrifugation, the clear supernatants were transferred into 5 mm NMR tubes. Spectra were acquired using a Bruker AVANCE 500 MHz NMR spectrometer (Bruker, Bremen, Germany) at 27 °C with a Carr–Purcell–Meiboom–Gill pulse sequence (CPMG; RD–90°–(τ–180°)_n_–acquire). The acquisition parameters were as follows: spectral width, 8278.146 Hz; dwell time, 60.40 µs; relaxation delay, 1 s; acquisition time, 3.95 s; and 16 scans.

Phase correction, baseline correction, and chemical-shift calibration were performed using Bruker TopSpin version 4.0.7. Spectra were evaluated over δH 0.0–12.0 ppm and referenced to TSP-d_4_ at δH 0.00 ppm. Spectral visualization and diagnostic-signal evaluation were performed using MestReNova software (version 14.1.2-25024; Mestrelab Research S.L., Santiago de Compostela, Spain). Spectral features were putatively annotated by comparing their ^1^H NMR chemical shifts and signal multiplicities with the Human Metabolome Database and published literature [48,49,50,51]. One diagnostic signal with minimal apparent overlap was selected for each feature. Signal responses were normalized to TSP-d_4_ and corrected for the contributing proton number and sample weight. For each feature, values were divided by the mean DL value, which was set to 1.00, and expressed as fold differences relative to DL. Because the acquisition conditions were not validated for quantitative NMR, the values were interpreted as relative signal intensities rather than absolute concentrations. Feature annotations remained tentative because they were not confirmed using authentic standards, 2D NMR, or LC–MS/MS.

### 4.3. Determination of Total Phenolic and Flavonoid Contents

The TPC and TFC of lychee pulp extracts were determined using colorimetric assays with slight modifications from previously reported methods [52]. For TPC analysis, the extract solution was mixed with Folin–Ciocalteu reagent, followed by the addition of sodium carbonate solution. The reaction mixture was incubated in the dark at room temperature to allow color development. Absorbance was measured at 765 nm using a UV–Vis spectrophotometer (Thermo Fisher Scientific, Waltham, MA, USA). Gallic acid was used as the reference standard, and the results were expressed as milligrams of gallic acid equivalents per gram of extract (mg GAE/g extract). For TFC determination, the extract was reacted sequentially with sodium nitrite (NaNO_2_), aluminum chloride (AlCl_3_), and sodium hydroxide (NaOH). After incubation, absorbance was recorded at 510 nm using the same UV–Vis spectrophotometer. Quercetin was used as the calibration standard, and the flavonoid content was expressed as milligrams of quercetin equivalents per gram of extract (mg QE/g extract).

### 4.4. Evaluation of Antioxidant Activities

The antioxidant activities of lychee pulp extracts were evaluated using DPPH radical scavenging, ABTS radical cation scavenging, and ferric reducing antioxidant power (FRAP) assays [52,53]. For the DPPH assay, the extract solution was mixed with DPPH reagent and incubated in the dark at room temperature. The decrease in absorbance resulting from radical scavenging activity was measured spectrophotometrically at 517 nm. The antioxidant activity was expressed as percentage inhibition relative to the control. For the ABTS assay, the ABTS•^+^ radical cation solution was freshly prepared and adjusted to the appropriate absorbance before use. The extract was added to the ABTS•^+^ solution and allowed to react for a fixed period. Absorbance was measured at 734 nm using a UV–Vis spectrophotometer (Thermo Fisher Scientific, Waltham, MA, USA), and the results were expressed as percentage inhibition. The FRAP assay was performed using freshly prepared FRAP reagent consisting of acetate buffer, TPTZ solution, and ferric chloride solution. After mixing the extract with the FRAP reagent and incubating at room temperature, absorbance was measured at 593 nm using the same spectrophotometer. The reducing power was calculated from a ferrous sulfate calibration curve and expressed as milligrams Fe^2+^ equivalents per gram of extract (mg Fe^2+^ eq/g extract).

### 4.5. In Vitro Inhibitory Activities Against Carbohydrate-Hydrolyzing Enzymes

The inhibitory activities of lychee pulp extracts against α-amylase and α-glucosidase were evaluated using colorimetric assays adapted from previously reported methods with slight modifications [53,54]. Extract solutions were prepared at concentrations of 0.5, 1, 10, 50, 100, and 200 mg/mL. All experiments were performed in triplicat.

For α-amylase inhibition, porcine pancreatic α-amylase (catalog no. A3176; Sigma-Aldrich, St. Louis, MO, USA) was used as the enzyme source. The extract solution was preincubated with the enzyme in 0.1 M phosphate buffer (pH 7.0) for 5 min, followed by the addition of 2-chloro-4-nitrophenyl-α-D-maltotrioside (CNPG3) as the substrate. The reaction mixture was incubated at 37 °C for 15 min, and absorbance was measured at 405 nm using a microplate reader (BioTek Instruments, Inc., Winooski, VT, USA). Acarbose served as the positive control and exhibited an IC_50_ value of 0.021 mg/mL.

For α-glucosidase inhibition, the extract solution was mixed with α-glucosidase from *Saccharomyces cerevisiae* (catalog no. G0660; Sigma-Aldrich, St. Louis, MO, USA) in 0.1 M phosphate buffer (pH 7.0). After preincubation for 5 min, p-nitrophenyl-α-D-glucopyranoside (pNPG) was added as the substrate, and the reaction mixture was incubated at 37 °C for 30 min. The release of p-nitrophenol was determined by measuring absorbance at 405 nm using the same microplate reader. Acarbose was used as the positive control, with an IC_50_ value of 0.702 mg/mL under the assay conditions employed in this study.

The percentage inhibition was calculated relative to the control reaction. Dose–response curves were constructed using GraphPad Prism (version 5.0; GraphPad Software, Inc., San Diego, CA, USA), and IC_50_ values were estimated by nonlinear regression analysis.

### 4.6. Cytotoxicity Evaluation by MTT Assay

The cytotoxicity of lychee pulp extracts was evaluated in EA.hy926 endothelial cells using the MTT assay, as previously described [55,56] with minor modifications. EA.hy926 cells were seeded into 96-well plates at a density of 1 × 10^4^ cells/well and allowed to attach overnight. The cells were subsequently treated with DL, AL, or ATAL extracts at concentrations of 3.125–200 µg/mL for 24 and 48 h.

Following treatment, MTT solution (5 mg/mL stock solution) was added to each well and incubated at 37 °C for 2 h. The culture medium was then removed, and the resulting formazan crystals were dissolved in dimethyl sulfoxide (DMSO). Absorbance was measured at 570 nm using a Synergy HT Multi-Mode Microplate Reader (BioTek Instruments, Inc., Winooski, VT, USA). Cell viability was expressed as a percentage relative to the untreated control.

### 4.7. Statistical Analysis

Data are presented as mean ± SD from three independent extraction replicates per treatment (*n* = 3). Technical measurements or wells from the same extract were averaged to obtain one value per independent extract before statistical analysis. Statistical analyses were performed using SPSS software (version 16.0; SPSS Inc., Chicago, IL, USA). Differences among DL, AL, and ATAL were evaluated using ANOVA, followed by Tukey’s honestly significant difference test when the omnibus ANOVA was significant. Mean differences, simultaneous 95% confidence intervals, exact omnibus *p*-values, and Tukey-adjusted pairwise *p*-values are reported in Tables S1 and S2. Statistical significance was defined as a two-sided *p* < 0.05.

For the cell-viability assay, technical wells were averaged within each independent experiment, and viability was normalized to the corresponding untreated control, which was set to 100%. Each extract–concentration condition was compared with 100% using a two-sided one-sample *t*-test. The resulting *p*-values were adjusted using the Holm procedure across the 21 extract–concentration comparisons within each exposure time. Pointwise 95% confidence intervals for the mean differences from 100% and Holm-adjusted *p*-values are presented in Table S3.

Exploratory multivariate analyses were performed using MetaboAnalyst 6.0 (https://www.metaboanalyst.ca). The data matrix comprised the relative signal intensities of 16 putatively annotated ^1^H NMR spectral features from nine independent extracts (three per treatment). Before principal component analysis, the data were log_2_-transformed, mean-centered, and autoscaled to unit variance. Hierarchical clustering heatmap visualization was performed using Euclidean distance and Ward’s linkage, with values displayed as feature-wise standardized relative signal intensities. TPC, TFC, antioxidant activities, and enzyme-inhibition data were not included in the multivariate analyses.

## 5. Conclusions

Aging and subsequent autoclave treatment modified the relative ^1^H NMR spectral-feature profiles of lychee pulp extracts and increased their total phenolic and flavonoid contents, antioxidant activities, and in vitro carbohydrate-hydrolyzing enzyme inhibition compared with dried lychee. PCA showed distinct processing-associated patterns among the putatively annotated spectral features. None of the extracts significantly reduced endothelial cell viability under the tested conditions. Overall, aging followed by autoclave treatment may be a useful approach for enhancing the phytochemical and in vitro functional properties of lychee-derived ingredients.

## Figures and Tables

**Table 1:** Putative annotation, diagnostic ^1^H NMR signals, and relative signal intensities of phytochemical spectral features in differently processed lychee extracts.

Phytochemical Class	Putatively Annotated Spectral Feature	Diagnostic ^1^H NMR Signals (δH, ppm)	Relative Signal Intensity (Fold of DL)			ANOVA *p*-Value
			DL	AL	ATAL	
Phenolic acids	Gallic acid	6.93 (d), 7.29 (d)	1.00 ± 0.03 ^c^	5.45 ± 0.04 ^a^	4.73 ± 0.12 ^b^	<0.001
	Caffeic acid	6.29 (d), 6.80 (d), 6.96 (dd), 7.03 (d), 7.55 (d)	1.00 ± 0.03 ^c^	4.60 ± 0.05 ^a^	4.07 ± 0.13 ^b^	<0.001
	*p*-Coumaric acid	6.47 (d), 6.91 (dd), 7.08 (s), 7.17 (d), 7.33 (dd)	1.00 ± 0.04 ^b^	6.34 ± 0.03 ^a^	6.46 ± 0.14 ^a^	<0.001
Flavan-3-ols	Catechin	2.50 (dd), 2.84 (dd), 3.97 (m), 4.56 (d), 5.85 (d), 5.92 (d), 6.71 (dd), 6.76 (d), 6.83 (d)	1.00 ± 0.01 ^b^	0.97 ± 0.04 ^b^	2.51 ± 0.02 ^a^	<0.001
	Epicatechin	2.77 (m), 2.91 (m), 4.32 (m), 4.95 (m), 6.09 (s), 6.12 (s), 6.93 (m), 7.04 (d)	1.00 ± 0.00 ^c^	12.31 ± 0.58 ^a^	5.18 ± 0.06 ^b^	<0.001
	Epigallocatechin gallate	2.85 (dd), 2.96 (dd), 3.30 (dt), 4.87 (s), 4.96 (s), 5.52 (s), 5.95 (s), 6.49 (s), 6.94 (s)	1.00 ± 0.02 ^c^	2.39 ± 0.07 ^b^	4.86 ± 0.16 ^a^	<0.001
Proanthocyanidins	Procyanidin B2	2.39 (d), 2.50 (d), 2.68 (s), 3.17 (s), 3.35 (s), 3.62 (s), 4.17 (s), 4.30 (s), 4.44 (s),4.75 (s), 4.92 (d), 5.72 (s), 5.80 (d), 6.53 (s), 6.62 (t), 6.99 (s), 7.99 (s), 8.70 (s)	1.00 ± 0.04 ^b^	0.86 ± 0.05 ^b^	7.44 ± 0.59 ^a^	<0.001
Flavanonol	Taxifolin	2.50 (ddd), 3.33 (s), 4.50 (ddd), 4.97 (d), 5.85 (d), 5.90 (d), 6.74 (d), 6.87 (d), 8.97 (d), 9.03 (d), 10.82 (s), 11.90 (s)	1.00 ± 0.21 ^b^	1.34 ± 0.12 ^b^	8.32 ± 0.35 ^a^	<0.001
Flavonols	Quercetin	6.19 (s), 6.41 (s), 6.89 (d), 7.53 (d), 7.55 (d), 7.68 (d)	1.00 ± 0.02 ^b^	0.91 ± 0.02 ^b^	5.61 ± 0.20 ^a^	<0.001
	Kaempferol	6.18 (s), 6.39 (s), 6.90 (t), 8.08 (t)	1.00 ± 0.04 ^c^	1.33 ± 0.03 ^b^	2.48 ± 0.15 ^a^	<0.001
Flavonoid glycosides	Rutin	1.38 (d), 3.49 (s), 3.70 (dd), 3.71 (m), 3.84 (dd), 4.05 (ddd), 4.12 (dd), 4.37 (dd), 4.97 (d), 5.18 (d), 6.09 (s), 6.71 (s), 6.93 (dd), 7.05 (dd)	1.00 ± 0.04 ^b^	0.46 ± 0.01 ^c^	1.39 ± 0.01 ^a^	<0.001
	Isoquercitrin	3.75 (dd), 3.84 (dd), 3.87 (m), 6.09 (s), 6.12 (s), 6.71 (s), 6.88 (d), 6.93 (d)	1.00 ± 0.04 ^b^	0.55 ± 0.01 ^c^	3.34 ± 0.07 ^a^	<0.001
	Quercetin 4′-glycoside	3.49 (t), 3.56 (m),3.75 (d), 3.85 (d), 6.09 (d), 6.12 (d), 6.51 (d), 6.93 (d), 7.04 (s)	1.00 ± 0.01 ^b^	0.73 ± 0.03 ^c^	3.67 ± 0.10 ^a^	<0.001
	Meloside	3.49 (dd), 3.63 (dd), 3.72 (d), 3.75 (dd), 3.80 (dd), 3.84 (dd), 3.85 (t), 4.05 (s), 4.12 (dd), 4.37 (s), 4.95 (s), 5.18 (d), 6.12 (s), 6.51 (s), 6.78 (d), 6.93 (d)	1.00 ± 0.00 ^b^	0.81 ± 0.03 ^b^	7.10 ± 0.14 ^a^	<0.001
Anthocyanins	Cyanidin	2.85 (dd), 3.49 (m), 3.64 (m), 3.75 (m), 4.05 (m), 4.12 (m), 4.24 (m), 4.36 (m), 4.95 (s), 5.16 (s), 6.50 (d), 6.70 (d), 6.75 (d), 6.90 (s), 6.93 (s), 6.94 (dd), 7.05 (m), 7.53 (t)	1.00 ± 0.02 ^b^	0.51 ± 0.02 ^c^	2.53 ± 0.04 ^a^	<0.001
	Delphinidin 3-glucoside	3.42 (ddd), 3.46 (dd), 3.63 (dd), 3.82 (t), 4.04 (d), 5.05 (d), 6.70 (d), 6.86 (d), 6.94 (s), 8.58 (s)	1.00 ± 0.05 ^b^	0.31 ± 0.02 ^c^	3.42 ± 0.01 ^a^	<0.001

Values are presented as the mean ± SD of three independent extraction batches (*n* = 3). For each putatively annotated spectral feature, the TSP-d_4_-normalized diagnostic signal intensity of each replicate was divided by the mean value of the corresponding DL group, which was set to 1.00. AL and ATAL values are therefore expressed as fold differences relative to DL. These values represent relative NMR signal intensities and not absolute metabolite concentrations. Statistical analyses were performed separately for each spectral feature using one-way analysis of variance (ANOVA), followed by Tukey’s honestly significant difference (HSD) test. Different superscript letters within the same row indicate significant differences among treatments (Tukey-adjusted *p* < 0.05). The reported *p*-values represent the overall treatment effects.

**Table 2:** TPC, TFC, DPPH radical scavenging activity, ABTS radical cation scavenging activity, and FRAP of lychee pulp extracts.

Parameters	DL	AL	ATAL	ANOVA *p*-Value
TPC (mg GAE/g extract)	0.53 ± 0.07 ^c^ (0.37 to 0.69)	8.39 ± 0.19 ^b^ (7.92 to 8.86)	9.07 ± 0.10 ^a^ (8.84 to 9.31)	<0.001
TFC (mg QE/g extract)	0.20 ± 0.03 ^c^ (0.11 to 0.27)	2.89 ± 0.07 ^b^ (2.72 to 3.06)	3.22 ± 0.05 ^a^ (3.09 to 3.35)	<0.001
DPPH (mg TE/g extract)	1.77 ± 0.28 ^c^ (1.07 to 2.47)	17.91 ± 1.60 ^a^ (13.92 to 21.90)	18.96 ± 1.64 ^a^ (14.88 to 23.04)	<0.001
ABTS (mg TE/g extract)	0.91 ± 0.28 ᶜ (0.21 to 1.61)	13.51 ± 1.93 ^b^ (8.72 to 18.29)	19.08 ± 1.38 ^a^ (15.65 to 22.51)	<0.001
FRAP (mg FeSO_4_/g extract)	1.01 ± 0.17 ^b^ (0.60 to 1.42)	20.60 ± 0.80 ^a^ (18.62 to 22.59)	19.74 ± 0.55 ^a^ (18.36 to 21.12)	<0.001

Values are presented as mean ± SD (*n* = 3 independent extractions), with 95% confidence intervals shown in parentheses. Each independently prepared extract was measured in technical triplicate, and the mean of the technical measurements was used as one extraction-level observation for statistical analysis. DPPH and ABTS radical scavenging activities were determined at an extract concentration of 10 mg/mL. Overall differences were evaluated using one-way ANOVA followed by Tukey’s HSD test. Different superscript letters within the same row indicate significant differences among extracts (adjusted *p* < 0.05). DL, dried lychee; AL, aged lychee; ATAL, autoclave-treated aged lychee.

## Data Availability

The original contributions presented in the study are included in the article. Further inquiries can be directed to the corresponding author.

## Supplementary Materials

The following supporting information can be downloaded at https://www.mdpi.com/article/10.3390/plants15152290/s1, Table S1: Omnibus one-way ANOVA and Tukey’s HSD pairwise comparisons of relative ^1^H NMR signal intensities among differently processed lychee extracts; Table S2: Omnibus one-way ANOVA and Tukey’s HSD pairwise comparisons of TPC, TFC, antioxidant activities, and carbohydrate-hydrolyzing enzyme inhibitory activities among lychee pulp extracts; Table S3: Comparisons of EA.hy926 endothelial cell viability following exposure to lychee pulp extracts against the corresponding normalized control; Figure S1: Hierarchical clustering heatmap of the relative 1H NMR spectral-feature profiles of lychee pulp extracts.

## Abbreviations

The following abbreviations are used in this manuscript:

^1^H NMR	Proton Nuclear Magnetic Resonance
TPC	Total phenolic content
TFC	Total flavonoid content
DL	Dried lychee extract
AL	Aged lychee extract
ATAL	Autoclave-treated aged lychee extract
IC_50_	Half maximal inhibitory concentration
DPPH	2,2-Diphenyl-1-picrylhydrazyl
ABTS	2,2′-Azino-bis(3-ethylbenzothiazoline-6-sulfonic acid)
FRAP	Ferric reducing antioxidant power
PCA	Principal component analysis
HMDB	Human Metabolome Database
