# Supplementary material for: Effects of Thermal Aging and Subsequent Autoclave Treatment on the Phytochemical Fingerprint, Antioxidant Capacity, and In Vitro Carbohydrate-Hydrolyzing Enzyme Inhibitory Activity of Lychee Pulp Extracts
Source: Plants (Basel). 2026 Jul 26;15(15):2290. doi: 10.3390/plants15152290 (PMC13468406; doi:10.3390/plants15152290)
Supplement: Supplementary file 1 [file plants-15-02290-s001.zip › Supplementary Figure S1_Hierarchical clustering heatmap.pdf]

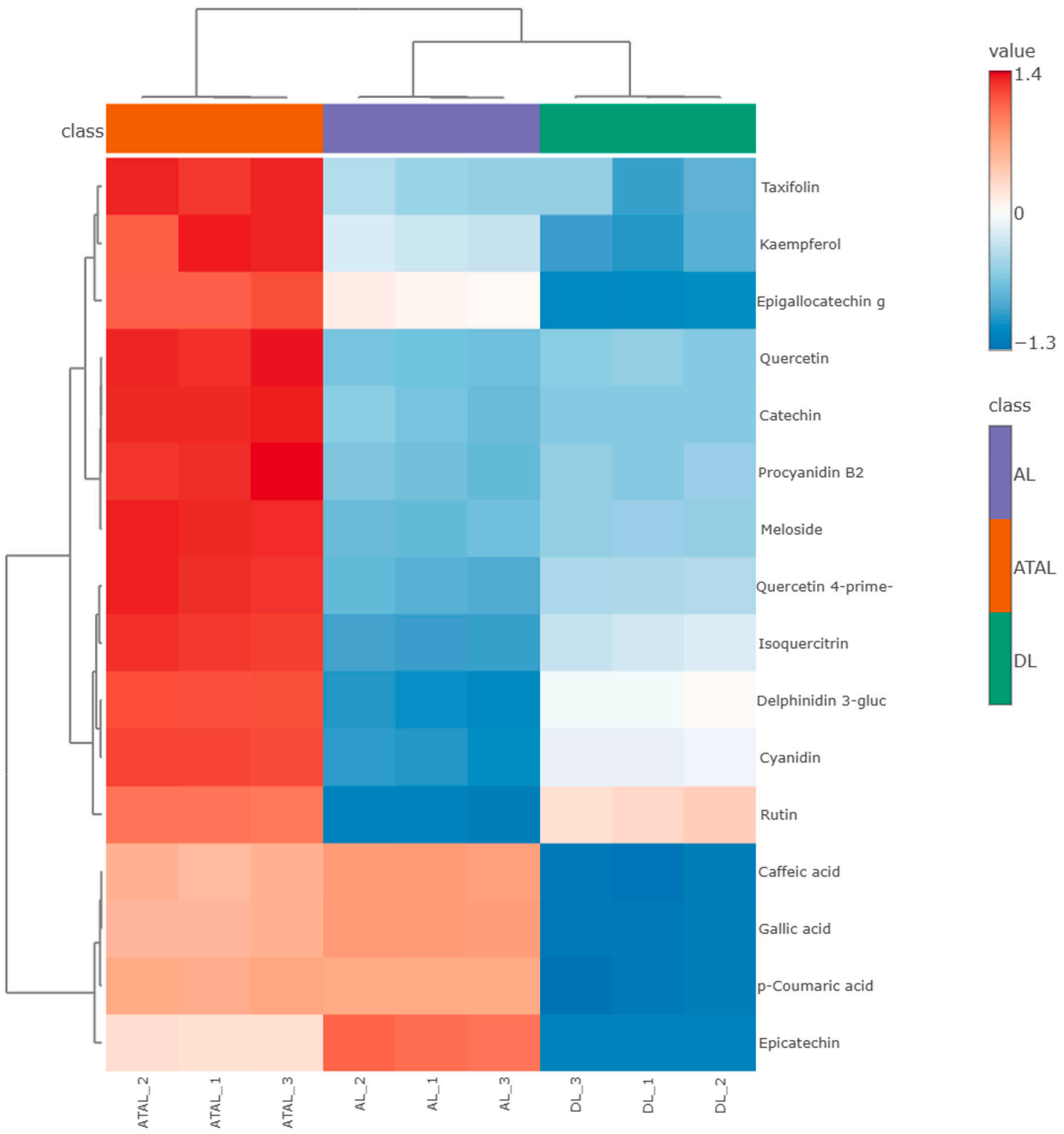

Figure S1. Hierarchical clustering heatmap of the relative ^{1}H NMR spectral-feature profiles of lychee pulp extracts. Relative signal intensities of 16 putatively annotated ^{1}H NMR spectral features were log_2-transformed and autoscaled. Hierarchical clustering was performed using Euclidean distance and Ward's linkage. Red and blue indicate relative signal intensities above and below the feature-wise mean, respectively. Each column represents one independent replicate (n = 3 per treatment). DL, dried lychee extract; AL, aged lychee extract; ATAL, autoclave-treated aged lychee extract.
