# Supplementary material for: Effects of Thermal Aging and Subsequent Autoclave Treatment on the Phytochemical Fingerprint, Antioxidant Capacity, and In Vitro Carbohydrate-Hydrolyzing Enzyme Inhibitory Activity of Lychee Pulp Extracts
Source: Plants (Basel). 2026 Jul 26;15(15):2290. doi: 10.3390/plants15152290 (PMC13468406; doi:10.3390/plants15152290)
Supplement: Supplementary file 1 [file plants-15-02290-s001.zip › Supplementary Table S1_Phytochemical.pdf]

Table S1. Omnibus one-way ANOVA and Tukey's HSD pairwise comparisons of relative ^{1}H NMR signal intensities among differently processed lychee extracts.

Putatively annotated spectral feature	F(2,6)	Omnibus p	Comparison	Mean difference	95% CI	Adjusted p
Gallic acid	3021.74	9.76×10^{-10}	DL–AL	-4.45	-4.64 to -4.26	2.49×10^{-7}
			DL–PSAL	-3.73	-3.92 to -3.54	2.49×10^{-7}
			AL–PSAL	0.72	0.53 to 0.91	5.74×10^{-5}
Caffeic acid	1774.68	4.81×10^{-9}	DL–AL	-3.60	-3.80 to -3.40	2.49×10^{-7}
			DL–PSAL	-3.07	-3.27 to -2.87	2.51×10^{-7}
			AL–PSAL	0.54	0.34 to 0.74	0.0004
p-Coumaric acid	3634.39	5.61×10^{-10}	DL–AL	-5.39	-5.61 to -5.16	2.49×10^{-7}
			DL–PSAL	-5.46	-5.68 to -5.23	2.49×10^{-7}
			AL–PSAL	-0.07	-0.30 to 0.16	0.6298
Catechin	2955.10	1.04×10^{-9}	DL–AL	0.03	-0.04 to 0.10	0.5148
			DL–PSAL	-1.51	-1.58 to -1.44	2.49×10^{-7}
			AL–PSAL	-1.54	-1.61 to -1.47	2.49×10^{-7}
Epicatechin	856.38	4.25×10^{-8}	DL–AL	-11.31	-12.16 to -10.46	2.60×10^{-7}
			DL–PSAL	-4.18	-5.02 to -3.33	1.32×10^{-5}
			AL–PSAL	7.13	6.29 to 7.98	7.13×10^{-7}
Epigallocatechin gallate (EGCG)	1095.82	2.04×10^{-8}	DL–AL	-1.39	-1.65 to -1.13	7.50×10^{-6}
			DL–PSAL	-3.86	-4.11 to -3.60	2.52×10^{-7}
			AL–PSAL	-2.47	-2.72 to -2.21	4.29×10^{-7}
Procyanidin B2	357.31	5.77×10^{-7}	DL–AL	0.14	-0.72 to 1.01	0.8696
			DL–PSAL	-6.44	-7.30 to -5.58	1.27×10^{-6}
			AL–PSAL	-6.58	-7.45 to -5.72	1.13×10^{-6}
Taxifolin	849.34	4.36×10^{-8}	DL–AL	-0.33	-0.95 to 0.28	0.2931
			DL–PSAL	-7.32	-7.93 to -6.70	2.83×10^{-7}
			AL–PSAL	-6.98	-7.60 to -6.37	2.99×10^{-7}
Quercetin	1637.75	6.11×10^{-9}	DL–AL	0.09	-0.19 to 0.38	0.6073
			DL–PSAL	-4.61	-4.90 to -4.32	2.50×10^{-7}
			AL–PSAL	-4.70	-4.99 to -4.42	2.50×10^{-7}
Kaempferol	229.23	2.16×10^{-6}	DL–AL	-0.33	-0.55 to -0.11	0.0093
			DL–PSAL	-1.48	-1.70 to -1.26	2.36×10^{-6}
			AL–PSAL	-1.15	-1.37 to -0.93	1.00×10^{-5}
Rutin	1140.94	1.80×10^{-8}	DL–AL	0.54	0.48 to 0.60	5.36×10^{-7}
			DL–PSAL	-0.39	-0.45 to -0.33	2.73×10^{-6}

Putatively annotated spectral feature	F(2,6)	Omnibus p	Comparison	Mean difference	95% CI	Adjusted p
			AL–PSAL	-0.93	-0.99 to -0.87	2.51×10^{-7}
			DL–AL	0.45	0.32 to 0.57	8.01×10^{-5}
Isoquercitrin	2749.22	1.30×10^{-9}	DL–PSAL	-2.34	-2.46 to -2.22	2.49×10^{-7}
			AL–PSAL	-2.79	-2.91 to -2.66	2.49×10^{-7}
			DL–AL	0.27	0.11 to 0.42	0.0045
Quercetin 4′-glycoside	2074.90	3.01×10^{-9}	DL–PSAL	-2.67	-2.83 to -2.52	2.49×10^{-7}
			AL–PSAL	-2.94	-3.09 to -2.79	2.49×10^{-7}
			DL–AL	0.19	-0.02 to 0.40	0.0771
Meloside	5247.33	1.87×10^{-10}	DL–PSAL	-6.10	-6.31 to -5.88	2.49×10^{-7}
			AL–PSAL	-6.29	-6.50 to -6.07	2.49×10^{-7}
			DL–AL	0.49	0.43 to 0.55	1.17×10^{-6}
Cyanidin	4996.05	2.16×10^{-10}	DL–PSAL	-1.53	-1.59 to -1.47	2.49×10^{-7}
			AL–PSAL	-2.02	-2.08 to -1.96	2.49×10^{-7}
			DL–AL	0.69	0.61 to 0.77	8.26×10^{-7}
Delphinidin 3-glucoside	7056.88	7.67×10^{-11}	DL–PSAL	-2.43	-2.52 to -2.35	2.49×10^{-7}
			AL–PSAL	-3.12	-3.21 to -3.04	2.49×10^{-7}

Mean differences were calculated as the mean relative signal intensity of the first group minus that of the second group. Exact omnibus p-values were obtained from one-way ANOVA. Pairwise p-values and simultaneous 95% confidence intervals were adjusted using Tukey’s honestly significant difference (HSD) procedure. For each putatively annotated spectral feature, the TSP-d_4-normalized diagnostic signal intensity was expressed relative to the mean value of the corresponding DL group, which was set to 1.00. These values represent relative NMR signal intensities and not absolute metabolite concentrations. DL, dried lychee extract; AL, aged lychee extract; PSAL, pressurized-steam-treated aged lychee extract.
