# Supplementary material for: Effects of Thermal Aging and Subsequent Autoclave Treatment on the Phytochemical Fingerprint, Antioxidant Capacity, and In Vitro Carbohydrate-Hydrolyzing Enzyme Inhibitory Activity of Lychee Pulp Extracts
Source: Plants (Basel). 2026 Jul 26;15(15):2290. doi: 10.3390/plants15152290 (PMC13468406; doi:10.3390/plants15152290)
Supplement: Supplementary file 1 [file plants-15-02290-s001.zip › Supplementary Table S2_TPC TFC & antioxidant.pdf]

Table S2. Omnibus one-way ANOVA and Tukey's HSD pairwise comparisons of TPC, TFC, antioxidant activities, and carbohydrate-hydrolyzing enzyme inhibitory activities among lychee pulp extracts.

Parameters	$F(2,6)$	Omnibus p	Comparison	Mean difference	95% CI	Adjusted p
TPC	4197.45	3.64×10^{-10}	DL–AL	−7.86	−8.18 to −7.55	8.80×10^{-10}
			DL–ATAL	−8.55	−8.86 to −8.23	5.34×10^{-10}
			AL–ATAL	−0.68	−1.00 to −0.37	0.0014
TFC	2841.12	1.17×10^{-9}	DL–AL	−2.70	−2.83 to −2.56	3.18×10^{-9}
			DL–ATAL	−3.03	−3.16 to −2.89	1.59×10^{-9}
			AL–ATAL	−0.33	−0.47 to −0.19	7.15×10^{-4}
DPPH	156.01	6.72×10^{-6}	DL–AL	−16.14	−19.48 to −12.79	1.49×10^{-5}
			DL–ATAL	−17.18	−20.53 to −13.84	1.03×10^{-5}
			AL–ATAL	−1.05	−4.39 to 2.30	0.6262
ABTS	137.01	9.84×10^{-6}	DL–AL	−12.60	−16.05 to −9.15	7.47×10^{-5}
			DL–ATAL	−18.18	−21.63 to −14.72	8.86×10^{-6}
			AL–ATAL	−5.58	−9.03 to −2.12	0.0061
FRAP	1128.31	1.86×10^{-8}	DL–AL	−19.59	−21.02 to −18.16	3.02×10^{-8}
			DL–ATAL	−18.73	−20.16 to −17.30	3.95×10^{-8}
			AL–ATAL	0.86	−0.57 to 2.29	0.2323
α-Amylase IC_{50} (mg/mL)	20.21	0.0022	DL–AL	117.83	51.34 to 184.33	0.0039
			DL–ATAL	120.73	54.24 to 187.23	0.0034
			AL–ATAL	2.90	−63.60 to 69.40	0.9902
α-Glucosidase IC_{50} (mg/mL)	418.76	3.60×10^{-7}	DL–AL	121.22	105.28 to 137.16	1.01×10^{-6}
			DL–ATAL	137.63	121.69 to 153.57	4.74×10^{-7}
			AL–ATAL	16.41	0.47 to 32.35	0.0447

Mean differences were calculated as the value of the first group minus that of the second group. Exact omnibus p-values were obtained from one-way ANOVA. Pairwise p-values and simultaneous 95% confidence intervals were adjusted using Tukey's HSD procedure. For enzyme inhibitory assays, a lower IC_{50} value indicates stronger inhibitory activity. DL, dried lychee; AL, aged lychee; ATAL, autoclave-treated aged lychee; IC_{50}, half-maximal inhibitory concentration.
