# Supplementary material for: Effects of Thermal Aging and Subsequent Autoclave Treatment on the Phytochemical Fingerprint, Antioxidant Capacity, and In Vitro Carbohydrate-Hydrolyzing Enzyme Inhibitory Activity of Lychee Pulp Extracts
Source: Plants (Basel). 2026 Jul 26;15(15):2290. doi: 10.3390/plants15152290 (PMC13468406; doi:10.3390/plants15152290)
Supplement: Supplementary file 1 [file plants-15-02290-s001.zip › Supplementary Table S3_Cell viability.pdf]

Table S3. Comparisons of EA.hy926 endothelial cell viability following exposure to lychee pulp extracts against the corresponding normalized control.
24 h exposure

Extract	Concentration (μg/mL)	Cell viability (% of control)	Mean difference vs control	95% CI of mean difference	t(2)	Exact p	Holm-adjusted p
DL	3.125	109.47 ± 8.97	9.47	-12.81 to 31.74	1.828	0.2090	1.0000
	6.25	109.00 ± 8.68	9.00	-12.56 to 30.55	1.795	0.2144	1.0000
	12.5	108.62 ± 9.83	8.62	-15.79 to 33.04	1.520	0.2679	1.0000
	25	109.22 ± 9.49	9.22	-14.35 to 32.79	1.683	0.2345	1.0000
	50	109.54 ± 10.06	9.54	-15.45 to 34.52	1.643	0.2422	1.0000
	100	105.73 ± 7.06	5.73	-11.80 to 23.27	1.407	0.2947	1.0000
	200	103.18 ± 9.02	3.18	-19.23 to 25.59	0.611	0.6036	1.0000
AL	3.125	103.09 ± 4.20	3.09	-7.35 to 13.52	1.272	0.3312	1.0000
	6.25	101.97 ± 1.76	1.97	-2.41 to 6.35	1.934	0.1929	1.0000
	12.5	103.93 ± 5.60	3.93	-9.98 to 17.84	1.216	0.3481	1.0000
	25	101.79 ± 4.62	1.79	-9.69 to 13.27	0.669	0.5721	1.0000
	50	103.34 ± 5.01	3.34	-9.10 to 15.78	1.155	0.3673	1.0000
	100	98.78 ± 5.40	-1.22	-14.64 to 12.20	-0.390	0.7343	1.0000
	200	98.81 ± 3.69	-1.19	-10.36 to 7.97	-0.560	0.6318	1.0000
ATAL	3.125	100.70 ± 4.88	0.70	-11.42 to 12.82	0.250	0.8258	1.0000
	6.25	102.29 ± 6.18	2.29	-13.06 to 17.65	0.642	0.5865	1.0000
	12.5	101.38 ± 9.23	1.38	-21.55 to 24.32	0.260	0.8195	1.0000
	25	99.67 ± 9.69	-0.33	-24.41 to 23.75	-0.059	0.9586	1.0000
	50	99.19 ± 7.71	-0.81	-19.97 to 18.35	-0.182	0.8720	1.0000
	100	102.01 ± 8.02	2.01	-17.92 to 21.94	0.434	0.7067	1.0000
	200	99.31 ± 9.41	-0.69	-24.07 to 22.68	-0.128	0.9101	1.0000

Table S3. Continued — 48 h exposure

Extract	Concentration (μg/mL)	Cell viability (% of control)	Mean difference vs control	95% CI of mean difference	t(2)	Exact p	Holm-adjusted p
DL	3.125	102.14 ± 5.35	2.14	-11.14 to 15.41	0.692	0.5604	1.0000
	6.25	100.66 ± 2.95	0.66	-6.68 to 8.00	0.385	0.7372	1.0000
	12.5	101.35 ± 7.15	1.35	-16.40 to 19.11	0.328	0.7742	1.0000
	25	104.72 ± 5.65	4.72	-9.33 to 18.77	1.446	0.2851	1.0000
	50	100.76 ± 13.43	0.76	-32.62 to 34.13	0.097	0.9313	1.0000
	100	100.02 ± 10.45	0.02	-25.94 to 25.97	0.003	0.9982	1.0000
	200	102.87 ± 9.16	2.87	-19.88 to 25.63	0.543	0.6414	1.0000
AL	3.125	101.45 ± 4.45	1.45	-9.61 to 12.52	0.566	0.6286	1.0000
	6.25	100.45 ± 5.96	0.45	-14.36 to 15.26	0.131	0.9078	1.0000
	12.5	101.45 ± 5.93	1.45	-13.29 to 16.19	0.423	0.7133	1.0000
	25	95.36 ± 1.79	-4.64	-9.09 to -0.19	-4.483	0.0463	0.9731
	50	95.39 ± 6.38	-4.61	-20.46 to 11.24	-1.250	0.3376	1.0000
	100	95.59 ± 3.97	-4.41	-14.27 to 5.45	-1.925	0.1941	1.0000
	200	93.15 ± 4.87	-6.85	-18.93 to 5.24	-2.437	0.1351	1.0000
ATAL	3.125	101.96 ± 11.13	1.96	-25.69 to 29.61	0.305	0.7892	1.0000
	6.25	98.19 ± 14.10	-1.81	-36.84 to 33.21	-0.223	0.8446	1.0000
	12.5	101.15 ± 10.56	1.15	-25.09 to 27.39	0.188	0.8679	1.0000
	25	100.57 ± 4.33	0.57	-10.17 to 11.32	0.230	0.8397	1.0000
	50	97.52 ± 10.37	-2.48	-28.24 to 23.28	-0.415	0.7187	1.0000
	100	98.25 ± 9.32	-1.75	-24.90 to 21.39	-0.326	0.7757	1.0000
	200	92.43 ± 3.41	-7.57	-16.03 to 0.89	-3.849	0.0614	1.0000

Values are presented as the mean ± SD of three independent experiments (n = 3). Four technical wells were averaged within each independent experiment before statistical analysis. Within each experiment, cell viability was normalized to the corresponding untreated control, which was set to 100%. Mean differences were calculated as the mean cell viability of the treated condition minus 100%. Two-sided one-sample t-tests were used to compare the experiment-level viability values with 100%. The reported 95% confidence intervals are pointwise t-based confidence intervals for the mean differences. Exact p-values were adjusted using the Holm procedure across the 21 extract–concentration comparisons within each exposure time. Statistical significance was defined as Holm-adjusted $p < 0.05$. DL, dried lychee extract; AL, aged lychee extract; ATAL, autoclave-treated aged lychee extract.
